# The Potential of Topical Therapy for Diabetic Wounds: A Narrative Review

**DOI:** 10.7759/cureus.36887

**Published:** 2023-03-29

**Authors:** Umme Salma Rangwala, Fatema Tashrifwala, Nikita N Egbert, Abuzar A Asif

**Affiliations:** 1 Internal Medicine, Mahatma Gandhi Memorial Medical College, Indore, IND; 2 Internal Medicine, University of Mauritius, Belle Rive, MUS; 3 Internal Medicine, Dr. D. Y. (Dnyandeo Yashwantrao) Patil Medical College, Hospital & Research Centre, Pune, IND; 4 Internal Medicine, University of Illinois College of Medicine Peoria, Peoria, USA

**Keywords:** topical agent, types 2 diabetes, type i diabetes mellitus, chronic wound healing, diabetic wound

## Abstract

The rising prevalence of diabetes mellitus brings with it a rise in the occurrence of several complications of the disease such as chronic non-healing wounds. Diabetics are more prone to developing chronic wounds due to complications like peripheral neuropathy, poor foot care, hyperglycaemia and peripheral vascular diseases. The aim of this review is to discuss the various imbalances in the cytokine environment of diabetic wounds and to explore the developments in their management with an emphasis on agents that may be used topically to aid the healing process of chronic wounds. A systematic search was conducted on Scopus, PubMed and Google Scholar and relevant articles were shortlisted. We conclude that increased blood sugar impairs most phases of wound healing in several ways. Supplementary therapy with either topical or systemic cytokines is shown to promote wound healing in a diabetic wound.

## Introduction and background

Diabetes mellitus (DM) is a chronic metabolic disease that is defined by long-standing hyperglycaemia, and is mainly classified based on pathophysiology into two groups - type 1 and type 2 DM. Type 1 DM (T1DM), also known as insulin-dependent DM, is an autoimmune disease characterized by the destruction of the pancreatic beta-islet cells and their subsequent failure to produce insulin. In new-onset T1DM, studies have demonstrated that the most common types of immune cells infiltrating the pancreatic beta-islet cells to cause autoimmunity are the cytotoxic CD8+ T-cells, macrophages, and more recently, the mast cells, with the degree of infiltration correlating with the severity of the disease. Type 2 DM (T2DM), which is the more common form of the disease, is caused by increased insulin resistance and in the later stages, a deficiency of insulin production due to pancreatic cell burnout. There are several contributors to the development of T2DM, particularly obesity. The excess accumulation of adipose tissue contributes to the insulin resistance that sets off the development of T2DM and a state of chronic inflammation [1,2].

According to the National Diabetes Statistics Report of 2020, it is estimated that in the USA, 37.3 million people have diabetes (11.3% of the US population), of which 8.5 million people (23.0% of adults) remain undiagnosed. The prevalence of prediabetes is 96 million people aged 18 years or older (38.0% of the adult US population) [3]. According to the International Diabetes Federation (IDF), the prevalence of DM worldwide is 463 million, and by 2045, this number is expected to reach 700 million [4].

Long-standing, poorly controlled DM brings with it several complications that are a consequence of the exposure of tissue to chronic hyperglycaemia; these complications can be categorized as microvascular and macrovascular. Microvascular complications include retinopathy, nephropathy and peripheral neuropathy, whereas macrovascular complications affect the cerebrovascular, cardiovascular and peripheral vascular systems [5]. 

The term ‘Diabetic foot’ is an umbrella term used to define one of the most common complications of DM non-healing wounds. It includes chronic wounds that take longer than three months to heal [6], diabetic ulcers, infection and gangrene. Its causation, which is otherwise multifactorial, can be attributed to microvascular complications like peripheral neuropathy and macrovascular complications like peripheral artery disease, for the sake of simplification. The surgeon T.D. Pryce in 1887 was the first to draw a connection between DM and foot ulceration [5,7]. Diabetics are reported to have a 25% lifetime risk of developing one of the most debilitating complications of the disease, which is chronic non-healing wounds and diabetic foot ulcers (DFU) in particular. It carries a 40% risk of leading to lower-limb amputations. In the USA, 67% of amputations and in the UK, 90% of amputations are caused by the complications of chronic wounds and DFU, making them the leading cause of non-traumatic lower-extremity amputations [4,8]. The inpatient costs associated with the management of DFU range from USD 20,000 to 40,000, while the outpatient personal costs range from USD 4,500 to 28,000. It is not just the hefty economic burden that is of note, but also the morbidity, mortality and psychosocial issues caused by DFU that warrant the need for further research into new modalities for DFU treatment [4,5].

The aim of this review is to discuss the various imbalances in the cytokine environment of diabetic wounds and to explore the developments in their management with an emphasis on agents that may be used topically to aid the healing process of chronic wounds. The use of platelet-derived growth factor (PDGF) is the only United States Food and Drug Administration (FDA)-approved growth factor for aiding the healing process of DM wounds [8]. This advancement signifies the scope of exploring a new therapeutic modality for the management of DM wounds, that is, topical therapy.

## Review

The skin is an organ of the human body with the largest surface area of 16,000-18,000 cm^2^ on average in adults. It serves as a mechanical barrier between the internal and external environments of our body and has neuroendocrine and immunologic capabilities. Cells such as the melanocytes, keratinocytes, Langerhans cells and sensory axons constitute the epidermis, which is the superficial layer of the skin. The deeper skin layer, the dermis, is composed of appendages like sebaceous glands and hair follicles, immune cells and the extracellular matrix that imparts structural support. Cutaneous innervation is by a complex system of autonomic and sensory nerve endings that are present in close proximity to the keratinocytes. The vascular supply arises from the subepidermal capillary network that forms a thermoregulatory shunt circulation regulated by the adrenergic sympathetic tone [9]. 

The process of acute wound healing

The normal process of wound healing is an intricately coordinated biological event that consists of four phases that progress linearly but may overlap: homeostasis, inflammation, proliferation and, finally, the remodeling phase.

Homeostasis is the first phase in the wound healing process, which is aimed at stopping bleeding by initiating localized vasoconstriction and sealing the disrupted skin barrier quickly and temporarily through platelet aggregation and activation. Tissue injury exposes the thrombogenic subendothelial collagen to various proteins and receptors on the surface of platelets; this results in the platelet plug formation that serves as a scaffold which will subsequently be stabilized by a fibrin mesh. The cutaneous peripheral nervous system is among the first to respond to a wound because the nociceptive receptors at the sensory nerve endings initiate and transmit action potentials to the spinal cord, which gives rise to the perception of the sensation of pain. These action potentials also activate the axonal reflexes that stimulate the release of neuropeptides like substance P (SP) and calcitonin gene-related peptide (CGrP) from sensory nerve endings. SP and CGrP promote vasodilation, increase vascular permeability and oedema formation and serve as chemoattractants for cells like mast cells, monocytes-macrophages and lymphocytes. Mast cell degranulation is stimulated by SP, which contributes to the development of the classic signs of acute inflammation - *Rubor, Calor, Dolor *and* Tumor* [9,10].

The acute inflammatory phase, lasting 2-5 days, is initiated in response to signals provided by injured tissue to inflammatory cells, neutrophils in particular. Several neutrophil receptors such as toll-like receptors (TLRs) react to the presence of damage-associated molecular patterns (DAMPs) like DNA fragments, peptides, ATP and uric acid released from injured cells, and pathogen-associated molecular patterns (PAMPs) released from wound-invading microbes [9]. This activates the process of neutrophil rolling, adhesion and migration into the wound site. Neutrophils kill the invading microbes by releasing secretory granules containing proteases such as cathepsin G, elastase, protease 3, myeloperoxidase, azurocidin, lysozyme, bacterial permeability-increasing protein, matrix metalloprotease 8, and collagenase-2, and by phagocytosis though the neutrophil extracellular traps (NETs) [10,11]. Circulating monocytes are recruited to the site of injury at around day 3 of wound healing where they transform into macrophages [12]. During this early wound healing phase, macrophages are of the pro-inflammatory (M1) type that releases cytokines like tumour necrosis factor-α (TNF-α), interleukin 6 (IL-6) and interleukin 1β (IL-1 β), and monocyte chemoattractant protein 1 (MCP-1); this eventually leads to the microbicidal activity of a large magnitude. The conclusion of the inflammatory phase of wound healing is heralded by the efferocytosis of neutrophils by M1 macrophages and the synthesis of miR-21, a microRNA, which leads to the switch from pro-inflammatory M1 to anti-inflammatory M2 macrophage type. The M2 phenotype increases the production of IL-10 and suppresses the production of TNF-α, IL-6 and IL-1β [9,13]. 

The proliferation phase begins 2-10 days after tissue injury and includes the processes of fibroplasia, re-epithelialization, neo-angiogenesis and peripheral nerve regeneration. The M2 macrophages are the dominant cell type in this phase. Granulation tissue, a highly vascularized but poorly differentiated soft tissue, is formed at the wound base and serves as a scaffold for the deposition of cells and extracellular matrix (ECM). Fibroblasts respond to growth factors like transforming growth factor β1 (TGF-β1), PDGF, epidermal growth factor (EGF) and fibroblast growth factor-2 (FGF-2) released mainly by M2 macrophages, by proliferating, promoting the production of inhibitors of metalloproteinases, migrating into granulation tissue and differentiating into myofibroblasts. Keratinocytes at the wound edge migrate into the granulation tissue after breaking free of their desmosome and hemidesmosome connections. As they fill the gap at the center of the wound, their cytoskeleton reorganizes to adopt a more flattened form to resemble the superficial layers of the epidermis more closely. Neo-angiogenesis is initiated by the proliferation and migration of microvascular endothelial cells in response to growth factors like vascular endothelial growth factor (VEGF), FGF, PDGF, TGF-β and angiopoietins [9,10,12].

The remodeling phase begins three weeks after injury and can last a few months. The granulation tissue gradually loses its vascular components, isolated and unorganized endothelial cells and myofibroblasts undergo apoptosis, and hydration- and structure-providing proteoglycans and glycosaminoglycan levels decline. Matrix metalloproteinases (MMPs) play a role in the replacement of type III collagen of the granulation tissue with type I collagen; this increases the tensile strength of the resultant scar [9,10].

The pathogenesis of chronic wounds in diabetes

Chronic wounds are characterized by persistent inflammation and disrupted reparative processes that do not abide by the expected timeline of acute wound healing. These can be categorized as diabetic ulcers, pressure ulcers and vascular ulcers - either venous or arterial. The imbalance in the levels of proteases and their inhibitors, along with increased production of reactive oxygen species (ROS), leads to the destruction of ECM. The immune cells recruited during the wound-healing process in a chronic wound differ from those present in the milieu of a normal acute wound, in that the growth factor receptor density on their cell membranes is reduced, and their ability to migrate and differentiate is also reduced. Other aberrancies in chronic wounds include the presence of an infectious biofilm, hyperproliferative epidermal wound edge, impeded re-epithelialization, elevated MMPs and impaired angiogenesis [6,11].

The effect that DM has on wound healing can be simply stated as follows - the acute, focused, phase of inflammation that occurs in the early stages of wound healing is not as robust as necessary and the later stages, which require a transition from pro-inflammatory to anti-inflammatory mediators, are dominated by the former. Its implications on wound healing can be appreciated by analysing the effect of hyperglycaemia and other DM-associated complications like neuropathy and peripheral vascular disease on various stages of and the cells involved in the process of wound healing.

What makes the skin of diabetics more susceptible to injury is a disrupted skin barrier and ineffective local bacterial invasion control. A decrease in lipid content and hydration that is influenced by poor local blood flow caused by microvascular and macrovascular changes of DM, and the formation of glycosylated collagen due to the accumulation of advanced glycation end products (AGEs) lead to the development of an inadequate mechanical skin barrier at baseline. Antimicrobial peptides (AMPs), which restrain the growth of pathogenic microbes on the skin surface and promote keratinocyte migration and angiogenesis, are decreased in DM chronic wounds [7,14,15].

Chronic hyperglycaemia overactivates the polyol and hexosamine pathways that result in sorbitol accumulation in nervous tissue and abnormal glycation of nerve cell proteins. This along with increased ROS lead to neuropathy (sensory, motor and autonomic). Diabetic neuropathy not only reduces the protective awareness towards a wound but also affects immune cell chemotaxis, neuropeptide and growth factor production and cell proliferation [13]. Atherosclerosis is a macrovascular complication of DM that contributes to poor wound healing by reducing blood flow in the wound bed [15].

The high levels of AGEs present in DM wounds reduce the migration of neutrophils across the endothelium by causing actin polymerization due to its binding with the AGEs receptor (RAGEs) present on the surface of neutrophils. This AGEs-RAGEs binding gives rise to a delayed but longer-lasting oxidative burst, thus having a detrimental effect on the opsonization and phagocytosis abilities of neutrophils [14,16]. There exists an imbalance in the M1/M2 levels during the early phases of wound healing, with pro-inflammatory M1 being deficient and anti-inflammatory M2 in excess. High AGEs lead to increased TNF-α in the later stages of wound healing. The switch from M1 to M2 is impaired due to insufficient upregulation of the peroxisome proliferator-activated receptor gamma (PPAR-γ). Under hyperglycaemic conditions, endothelial cells and fibroblasts secrete abnormally high levels of pro-inflammatory cytokines like MCP-1, TNF-α, IL-8 and MMPs, and undergo increased cellular dysfunction and apoptosis [14].

Potential topical treatments for DM wounds

This section discusses the molecular targets for topical therapy that are involved in various phases of the wound-healing process.

A major contributor to the healing impairment in diabetic wounds is the presence of a high level of pro-inflammatory cytokines like IL-6, IL-1β and TNF-α, which leads to persistent inflammation. A study by Liu et al. [17] illustrates the pro-apoptogenic effect of wound-secreted TNF-α on fibroblast populations in diabetic wounds. The intervention with a TNF-α-specific inhibitor, for example, infliximab, during wound healing significantly reduced caspase-3 activity and fibroblast apoptosis by almost 50%. This further proves the theory that DM is characterized by a pro-inflammatory cytokine spillover, particularly of TNF-α, IL-1 and IL-6 [7].

IL-10 is an anti-inflammatory mediator that attenuates the inflammatory response. It is paradoxically increased during the acute, inflammatory phase of diabetic wounds, which in turn causes a cascade of reductions in TLR signaling and proinflammatory cytokine production, delaying macrophage and leukocyte responses, and underlies the healing impairment in diabetic wounds. This is the basis of anti-IL-10 strategies which have therapeutic potential if added topically after surgical debridement, which resets chronic wounds into acute fresh wounds [18]. However, IL-10, which is vital in the proliferation and remodeling phases, is deficient during these very phases of DM wound healing. Therefore, methods that increase IL-10 expression such as highly purified eicosapentaenoic acid, nano-micelles of gene delivery vector N-acyl low-molecular-weight chitosan, and topical curcumin (a derivative of turmeric) present interesting options to improve DM wound healing when used in later stages [1]. 

TGF-β acts as a chemokine for cells of acute inflammation and stimulates fibroblasts to synthesize ECM while reducing their MMPs, which destroy collagen. Its level peaks in a biphasic manner, once during the inflammatory phase and later during the proliferation phase. Therefore, methods to increase TGF-β such as surgical debridement, which utilizes the principle that exposure to fresh serum, as occurs when a fresh wound is inflicted, bathes the skin in TGF to promote re-epithelialization or the use of nitric oxide that activates the latent form of TGF-β may be used to aid DM wound healing [1,19]. Components in cold atmospheric plasma such as growth factors, like FGF-2 and VEGF, and interleukins mediate granulation, vascularization and reepithelization in the process of healing in a diabetic foot [20]. 

AMPs, which regulate the skin flora and suppress the growth of pathogens, such as cathelicidin and human beta-defensin, improve wound healing by increasing keratinocyte migration. AMPs can be incorporated into hydrogels, nanoparticles and nanopolymers to be delivered directly to the wound [15]. 

A study by Lima et al. [21] reported that the proteins involved in the early actions of insulin are downregulated in the wounds of diabetic rats. The topical application of insulin to wounds resulted in the activation of the IR/IRS-1,2/AKT pathways, and this induced the production of VEGF and stromal cell-derived factor 1 (SDF-1), which increased angiogenesis, thereby improving diabetic wound healing. 

Myofibroblasts are essential for the contraction of the wound, which is a vital step in the formation of granulation tissue. It differentiates from the fibroblasts and contains microfilaments that function as muscle fibers by attaching to the desmosomes present along the wound edges. Insulin-like growth factor 1 (IGF-1) stimulates this process but is not present in sufficient levels in a diabetic wound. Supplementing the wound with IGF-1 may aid diabetic wound healing [22]. 

Although it is well known that mast cells are primarily involved in initiating allergic reactions, there is increasing evidence that they also play a role in wound healing and the homeostasis of the skin barrier. Wounds in diabetic mice pre-treated with a mast cell membrane stabilizer like disodium cromoglycate (DSCG) transformed macrophages from the M1 pro-inflammatory phenotype into the M2 regenerative phenotype, demonstrating that wound healing may be improved by the presence of intact non-degranulated mast cells. An indole carboxamide type of mast cell stabilizer, MCS-01, was developed to be topically delivered by alginate bandages. The topical application of MCS-01 had a similar effect to the systemic delivery of DSCG in that it reduced mast cell degranulation in diabetic mouse skin and accelerated wound healing. This suggests the potential for topical mast cell stabilizers in DM wound healing [2].

**Figure 1:**
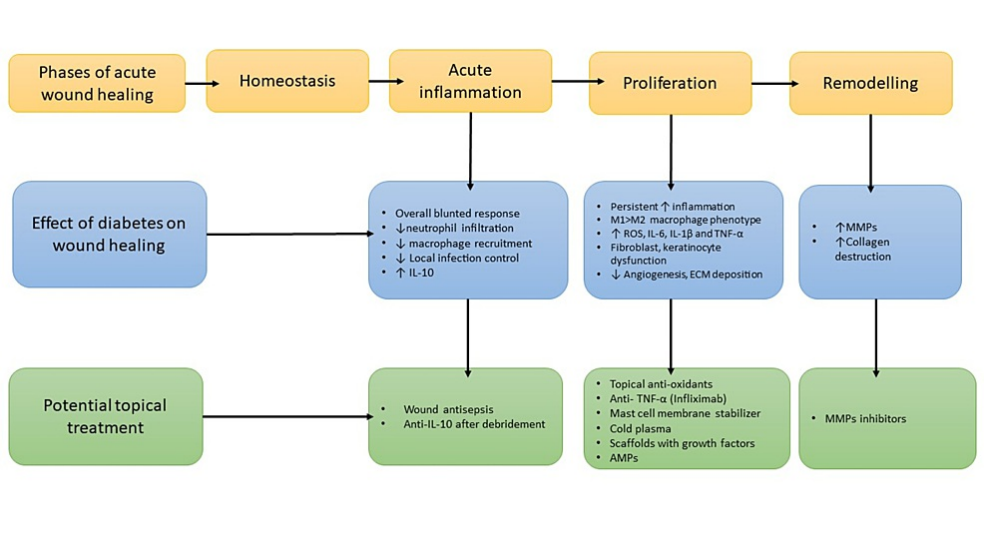
Article summary IL, interleukin; M1, pro-inflammatory macrophage; M2, anti-inflammatory macrophage; ROS, reactive oxygen species; TNF-α, tumour necrosis factor alpha; ECM, extracellular matrix; MMPs, matrix metalloproteinases; AMPs, antimicrobial peptides.

DM complications like hyperglycaemia interfere with the proliferation phase of wound healing, leading to decreased ECM deposition. This can be mitigated by using scaffolds that resemble the naturally occurring ECM structure and provide a framework for the migration and deposition of cells. Synthetic or naturally derived scaffolds impregnated with growth factors like EGF, basic fibroblast growth factor, keratinocyte growth factor, VEGF, IGF-1 and PDGF can provide a substrate for improved DM wound healing [23,24]. A summary of the discussions in this article is depicted in Figure 1. 

## Conclusions

From our review, we see how persistent hyperglycaemia impacts wound healing. Exposure to high blood sugar over a short period of time impacts the chronic phase of wound healing, whereas exposure to a comparatively lower blood sugar over prolonged periods of time impacts the acute phase. Since patients suffering from DM are immunocompromised and more susceptible to bacterial and fungal infections, a diabetic wound needs to be treated immediately and requires a multipronged strategy that must be inclusive of topical cytokines, systemic antimicrobials along with tight glycemic control. Pharmacological compliance is defined as the failure to take more than two doses of anti-diabetic medication over the last 15 days. The research found that adherence to oral anti-diabetic agents ranged from 36% to 93% across studies and that adherence to insulin was ∼63%. The adherence to topical therapy in patients of DM is self-reported to be around 50.5%. Through our study, we find that topical cytokine and interleukin therapy, used alongside antibiotic/antifungal therapy, decreases the wound healing time in DM wounds. Witnessing such encouraging results would in turn increase the compliance of patients with anti-diabetic medications. The scope of our article is to discuss only the topical regimens of cytokine and interleukin therapy and as such the adverse effect of such therapy is limited to rash, mild irritation and dermatitis. However, systemic therapy is known to cause nausea, vomiting and low blood pressure. In this background, we note that the main challenge to implementing topical therapy is the associated cost since this therapy is relatively expensive as compared to traditional regimens. More research is required to assess the efficacy and potency of topical cytokine and interleukin therapy in DM wounds as well as other cost-effective options. Further research is required to study the available treatment modalities for a diabetic wound with respect to possible drug therapies, the cytokine environment of the wound and the side effects of such treatments.
